# Using drug exposure for predicting drug resistance – A data-driven genotypic interpretation tool

**DOI:** 10.1371/journal.pone.0174992

**Published:** 2017-04-10

**Authors:** Alejandro Pironti, Nico Pfeifer, Hauke Walter, Björn-Erik O. Jensen, Maurizio Zazzi, Perpétua Gomes, Rolf Kaiser, Thomas Lengauer

**Affiliations:** 1 Department of Computational Biology and Applied Algorithmics, Max-Planck-Institut für Informatik, Saarbrücken, Germany; 2 Medizinisches Infektiologiezentrum Berlin, Berlin, Germany; 3 Medizinisches Labor Stendal, Stendal, Germany; 4 Clinic for Gastroenterology, Hepatology, and Infectiology, University Clinic of Düsseldorf, Düsseldorf, Germany; 5 Department of Medical Biotechnology, University of Siena, Siena, Italy; 6 Laboratorio de Biologia Molecular, LMCBM, SPC, HEM - Centro Hospitalar de Lisboa Ocidental, Lisbon, Portugal; 7 Centro de Investigacao Interdisciplinar Egas Moniz (CiiEM), Instituto Superior de Ciencias da Saude Sul, Caparica, Portugal; 8 Institute for Virology, University Clinic of Cologne, Cologne, Germany; Kyushu University, JAPAN

## Abstract

Antiretroviral treatment history and past HIV-1 genotypes have been shown to be useful predictors for the success of antiretroviral therapy. However, this information may be unavailable or inaccurate, particularly for patients with multiple treatment lines often attending different clinics. We trained statistical models for predicting drug exposure from current HIV-1 genotype. These models were trained on 63,742 HIV-1 nucleotide sequences derived from patients with known therapeutic history, and on 6,836 genotype-phenotype pairs (GPPs). The mean performance regarding prediction of drug exposure on two test sets was 0.78 and 0.76 (ROC-AUC), respectively. The mean correlation to phenotypic resistance in GPPs was 0.51 (PhenoSense) and 0.46 (Antivirogram). Performance on prediction of therapy-success on two test sets based on genetic susceptibility scores was 0.71 and 0.63 (ROC-AUC), respectively. Compared to geno2pheno_[resistance]_, our novel models display a similar or superior performance. Our models are freely available on the internet via www.geno2pheno.org. They can be used for inferring which drug compounds have previously been used by an HIV-1-infected patient, for predicting drug resistance, and for selecting an optimal antiretroviral therapy. Our data-driven models can be periodically retrained without expert intervention as clinical HIV-1 databases are updated and therefore reduce our dependency on hard-to-obtain GPPs.

## 1. Introduction

Prolonged chemotherapy against the *human immunodeficiency virus type 1* (HIV-1) bears the risk of selection of resistant viral strains, ultimately leading to therapy failure [1–6]. Once a drug-resistant HIV-1 variant has been selected in a host, it can be transmitted to another host [6,7]. Furthermore, drug-resistant viral variants are permanently archived in the body of the host and can promptly reemerge if drug pressure conveys them a competitive advantage to other viral variants [8]. In order to prevent premature therapy failure, the susceptibility of an HIV-1 variant to available antiretroviral drugs can be measured phenotypically or genotypically [4,9–12]. Due to the high cost, limited accessibility and high turnaround time of phenotypic resistance assays, genotypic resistance determination has become the standard of care [4,9]. Phenotypic resistance assays afford direct, quantitative resistance assessments that take into account resensitizing mutations [13], as well as complex mutational patterns [14]. However, certain drugs show significantly decreased *in-vivo* efficacy at very low *in-vitro* susceptibility changes which are close to the inherent variability of the phenotypic assay [15]. Furthermore, viral strains with mutations that do not directly cause resistance, but are strongly associated with the emergence of drug resistance, may be deemed susceptible by *in-vitro* phenotypic drug-resistance assays. If the respective drugs are taken by patients harboring these strains, resistant variants will promptly emerge and compromise virologic response to therapy [16].

Determination of genotypic resistance is performed by sequencing the viral genes coding for the targets of antiretroviral drugs, and subsequently interpreting the resulting nucleotide sequence [12]. A handful of tools exist for interpreting HIV-1 genotypes with respect to drug resistance. Drug-resistance mutation tables list amino acid mutations that confer resistance to antiretroviral drugs [14,17]. Rules-based genotypic interpretation systems score an HIV-1 genotype according to a set of rules defined by experts. The score for each drug is subsequently discretized into two to five categories indicating increasing levels of resistance [18,19]. Data-driven genotypic interpretation systems rely on statistical models of drug resistance for interpreting an HIV-1 genotype. These models are trained on sets usually containing genotype-phenotype pairs (GPP) [20,21] generated with *in vitro* phenotypic assays, and can thus potentially inherit their advantages and disadvantages.

HIV-1 substitutions resulting from chemotherapy are frequently divided in two groups: major drug-resistance mutations and minor drug resistance mutations, which can also occur as natural polymorphisms [14,17,22–26]. While there is no consensus on the definition of these two groups of mutations, in the following, we list the defining criteria that tend to be used. Major drug resistance mutations are frequently present in viral genotypes from patients failing antiretroviral therapy, and appear very rarely in HIV-1 genotypes from therapy-naïve patients. In fact, detection of such mutations in drug-naïve patients is currently interpreted as transmission of a resistant variant from patients who have failed therapy. By themselves, major drug-resistance mutations can either be directly responsible for drug resistance, or be informative markers for drug resistance. The implications of a mutation with respect to drug resistance can be investigated through site-directed mutagenesis with subsequent phenotypic resistance testing of the produced viral variant [27]. Minor drug resistance mutations tend to be polymorphic, and do not cause drug resistance by themselves, although they may further decrease susceptibility to a drug in combination with major drug resistance mutations and / or compensate for decreased replicative capacity resulting from selection of major mutations. In population genetics, a polymorphism is defined as a substitution that is present in more than one percent of the population [26,28]. The role HIV-1 polymorphisms play in chemotherapeutic success remains controversial [23,24,29–31]. Certain polymorphisms may tend to accumulate during chemotherapy while also being present in drug-naïve patients, albeit with a reduced frequency [22,23]. Polymorphisms present at baseline may influence the drug susceptibility of an HIV-1 variant [26,30–32]. Differential polymorphism distribution among HIV-1 subtypes has been observed, however, significant implications for drug susceptibility only seem to originate from intra-subtype variability as opposed to inter-subtype variability [24,25,32]. The most convincing explanation for the subtype-specific distribution of natural polymorphisms seems to be the existence of subtype-specific resistance pathways rather than subtype-specific propensity for selecting drug resistance [32].

Before drug resistance assays became available, treatment history was frequently used for the selection of new drug regimens [33]. Nowadays, new drug regimens are sometimes selected on the basis of treatment history when no drug resistance test is available. Indeed, statistical models that use treatment history in place of the genotype for predicting the success of antiretroviral therapy have been reported to be comparable to those of models that use the genotype (and do not use therapy history) [34–37]. However, to our knowledge, these methods have not yet found their way into clinical practice. In our experience, in today’s settings using therapy history as a proxy for genotype incurs substantial loss of predictive power. At the same time, a statistically significant increase in performance can be achieved by simultaneously using treatment history and the genotype for predicting the success of antiretroviral therapy [34,38–43].

**Fig 1:**
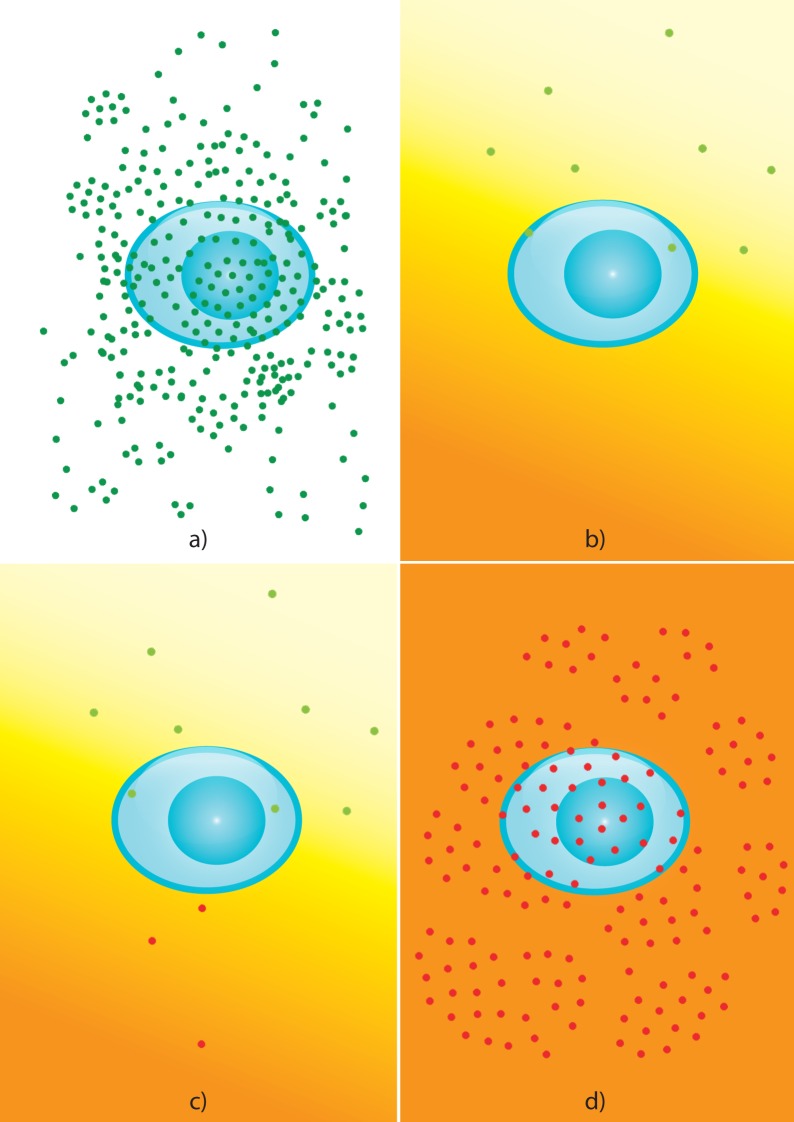
Relationship between drug exposure, drug resistance, and therapeutic success. a) Prior to drug exposure, the virus typically does not carry drug-resistance mutations. In the absence of drug pressure, drug-susceptible virus can replicate at high titers (dark-green viral particles). b) If drug susceptibility is given, antiretroviral therapy frequently leads to the suppression of viral replication, which is a prerequisite for therapeutic success. While antiretroviral therapy is administered, however, drug concentrations fluctuate over the dosing interval and may vary within the different body compartments (orange-yellow gradient). This can give rise to sub-inhibitory concentrations in some compartments (light-yellow area in gradient), resulting in the selection of mutations that confer to the virus a selective advantage in the presence of the drug (light-green viral particles). These mutations need not result in virological therapy failure, since they may not enable the virus to replicate at high drug concentrations. c) Recurrence of sub-inhibitory drug concentrations can ultimately select for mutations that enable the virus to replicate even at the highest drug concentrations (red viral particles). d) The selection of drug-resistant virus leads to virological therapy failure: the virus replicates at high titers in spite of antiretroviral therapy.

Drug exposure can be predicted from genotype since the virus acquires mutations as a result of being exposed to a drug. These mutations encompass but are not limited to drug-resistance mutations. Thus, while some of these mutations may indicate clinically relevant drug resistance, others may also solely indicate that the virus has changed as a result of drug exposure. As drug susceptibility is a prerequisite for the success of antiretroviral therapy, the detection of drug exposure may pose a risk for therapeutic success (Fig 1). Reporting drug exposure from genotype is relevant if either no established resistance-associated mutations are detectable and / or in cases in which no treatment-history information is available. In this work, we present statistical models that use HIV-1 genotypes to produce predictions of drug-exposure that are correlated with both therapeutic history and drug resistance. We have developed our method in close contact with prospective users. From the resulting experience, we expect the method to provide a significant clinical advance in bioinformatics-based therapy-success prediction.

Note that this article is largely based on another publication from which we amply quote [44].

## 2. Results

We trained models for predicting whether an HIV-1 variant had been previously exposed to a certain drug. One or two models were trained for each of the drugs considered in this study (Methods). Specifically, Exposure models were trained with HIV-1 sequences and information on drug exposure. The development sets of ExposurePheno models included genotype-phenotype pairs (GPPs) in addition to the data included in Exposure models. Since a sufficient number of HIV-1 sequences with information on drug-exposure was not available for all drugs, Exposure models could not be trained for all drugs. Additionally, we trained a model for discriminating between HIV-1 sequences from treatment-naïve patients and HIV-1 sequences from treatment-experienced patients. In the following, we refer to a number of datasets that we used for training and validating our models. For the comfort of the reader, we summarize the contents of each of these datasets in Table 1. Furthermore, we depict the relationships of each of the datasets in Figure A in S1 File.

**Table 1:** Dataset cheat sheet.

Dataset	Description	Input Variables	Target Variables
PRRT	Protease and reverse-transcriptase sequences from the EIDB and the LANLSD, along with the drug compounds previously used by the patient at the time of sequencing.	Sequence of protease and reverse transcriptase	Binary drug-exposure label for each protease inhibitor or reverse-transcriptase inhibitor
IN	Integrase sequences from the EIDB and the LANLSD, along with the drug compounds previously used by the patient at the time of sequencing.	Sequence of integrase	Binary drug-exposure label for each integrase inhibitor
TP	Past drug compounds and sequences in PRRT and in IN that were obtained during therapy pause.	Sequence of protease and reverse-transcriptase or integrase	Binary drug-exposure label for each drug
T_PRRT_	Test set of protease and reverse-transcriptase sequences and drug-exposure information.	Sequence of protease and reverse transcriptase	Binary drug-exposure label for each protease inhibitor or reverse-transcriptase inhibitor
T_IN_	Test set of integrase sequences and drug-exposure information.	Sequence of integrase	Binary drug-exposure label for each integrase inhibitor
D_PRRT_	Development set of protease and reverse-transcriptase sequences and drug-exposure information.	Sequence of protease and reverse transcriptase	Binary drug-exposure label for each protease inhibitor or reverse-transcriptase inhibitor
D_IN_	Development set of integrase sequences and drug-exposure information.	Sequence of integrase	Binary drug-exposure label for each integrase inhibitor
EuResistTCE	Test set of TCEs. Each TCE contains a protease and reverse-transcriptase baseline sequence, the drug compounds that were used in the therapy, and a label indicating therapeutic success or failure.	Baseline protease and reverse-transcriptase sequence for therapy	Binary therapy-success label
EuResistTCE_TP_	Test set of TCEs whose baseline sequences were obtained during a therapy pause. Each TCE contains a protease and reverse-transcriptase baseline sequence, the drug compounds that were used in the therapy, and a label indicating therapeutic success or failure.	Baseline protease and reverse-transcriptase sequence for therapy	Binary therapy-success label
HIVdbExposure	Test set of protease and reverse-transcriptase sequences and drug-exposure information.	Sequence of protease and reverse transcriptase	Binary drug-exposure label for each protease inhibitor or reverse-transcriptase inhibitor
HIVdbTCE	Test set of TCEs. Each TCE contains a protease and reverse-transcriptase baseline sequence, the drug compounds that were used in the therapy, and a label indicating therapeutic success or failure.	Baseline protease and reverse-transcriptase sequence for therapy	Binary therapy-success label
Pheno	Dataset of GPPs.	Protease, reverse-transcriptase or integrase sequence	Resistance factors for different drugs
T_Pheno_	Test set of GPPs.	Protease, reverse-transcriptase or integrase sequence	Resistance factors for different drugs or resistance categories
D_Pheno_	Development set of genotype-phenotype pairs.	Protease, reverse-transcriptase or integrase sequence	Resistance factors for different drugs or resistance categories
Naïve_PRRT_	Dataset of protease and reverse-transcriptase sequences from treatment-naïve patients without TDR mutations.	Sequence of protease and reverse transcriptase	None
Naïve_IN_	Dataset of integrase sequences from treatment-naïve patients without TDR mutations.	Sequence of integrase	None
Exposure_*drug*_	Cross-validation / development set for the compound *drug*. These datasets include sequences with drug exposure information but not GPPs.	Protease, reverse-transcriptase or integrase sequence	Binary drug-exposure label for *drug*
Exposure_naïvePRRT_	Cross-validation /development set for models discriminating sequences from treatment-exposed and treatment-naïve patients.	Protease and reverse-transcriptase sequence	Binary label indicating whether sequence was obtained from therapy-naïve patient
ExposurePheno_*drug*_	Cross-validation / development set for the compound *drug*. These datasets include sequences with drug exposure information and GPPs.	Protease, reverse-transcriptase or integrase sequence	Binary label indicating exposure or resistance to *drug*. Note that this label does not distinguish between drug exposure and drug resistance

In the table above, the names of the datasets used in this study are tabulated along with a short description of their contents. The datasets are shown in order of appearance in *Methods*. Above, the term *sequences* refers to HIV-1 nucleotide sequences.

EIDB: EuResist Integrated Database; GPP: genotype-phenotype pair; LANLSD: Los Alamos National Laboratory Sequence Database; TCE: Therapy-Change Episode; TDR: transmitted drug resistance.

### 2.1. Dataset preparation

**Fig 2:**
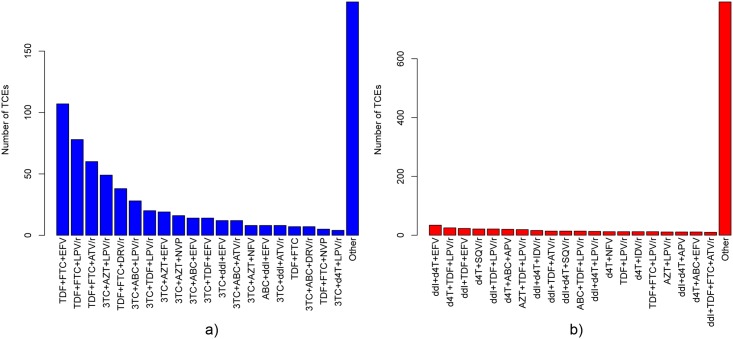
Drug-combination counts for therapies in EuResistTCE and HIVdbTCE. The frequencies of the 20 most-frequent drug combinations in EuResistTCE (a) and HIVdbTCE (b) datasets are displayed above. 3TC: lamivudine, ABC: abacavir, AZT: zidovudine, d4T: stavudine, ddI: didanosine, FTC: emtricitabine, TDF: tenofovir, EFV: efavirenz, NVP: nevirapine, APV: amprenavir, ATV: atazanavir, DRV: darunavir, IDV: indinavir, LPV: lopinavir, NFV: nelfinavir, SQV: saquinavir.

Prior to alignment, 48,666 nucleotide sequences with information on drug exposure were extracted from the EuResist Integrated Database (EIDB; http://www.euresist.org) [45] and aligned to reference sequences for the viral protease, reverse-transcriptase, and integrase. The alignment procedure yielded 38,754 sequences for protease and reverse-transcriptase (assigned to the PRRT dataset) and 6,214 integrase sequences (assigned to the IN dataset). PRRT and IN were further complemented with 36,774 and 5,262 sequences from therapy-naïve patients (short: therapy-naïve sequences), respectively, from the Los Alamos National Laboratory Sequence Database (LANLSD; http://www.hiv.lanl.gov/). The number of sequences in PRRT was reduced to 75,239 sequences after excluding sequences with more than 10% undetermined residues. After removal of duplicate sequences, PRRT included a total of 70,304 sequences (approximately 93% of the initially included sequences). The number of sequences in IN was reduced to 7,076 after excluding sequences with more than 10% undetermined residues. After duplicate removal, 5,523 sequences (approximately 48%) were left in IN. The number of sequences per subtype for PRRT and IN can be seen in Table 2. Sequences in PRRT and IN were randomly assigned either to the development sets D_PRRT_ and D_IN_, respectively, or to the test sets T_PRRT_ and T_IN_, respectively (Methods). Two additional test sets were created, TP and HIVdbExposure. TP contains sequences from T_PRRT_ and T_IN_ which were obtained during therapy pauses. HIVdbExposure was created from the treatment-change episode (TCE) repository in the HIV Drug Resistance Database (HIVdb) [14,46]. It contains nucleotide sequences and lists the sets of drug compounds that had been used by the patient before the sequence was obtained. The distribution of subtypes per sequence in HIVdbExposure can be seen in Table 2. Table 3 shows the number of sequences in datasets D_PRRT_, D_IN_, T_PRRT_, T_IN_, TP, and HIVdbExposure by drug exposure. In D_PRRT_, 37,557 sequences were therapy-naïve, of which 3,757 (10.0%) present transmitted drug resistance (TDR) [7]. A total of 1,917 sequences in D_IN_ are therapy-naïve, of which 48 (approximately 2%) present TDR. Note that the duplicate removal procedure eliminated substantially more sequences from IN than from PRRT. T_PRRT_ contains 2,056 therapy-naïve sequences, among which 219 (approximately 11%) present TDR, while T_IN_ contains 154 therapy-naïve sequences with 3 (approximately 2%) presenting TDR. We applied the definition of the EuResist Standard Datum [40] to clinical HIV data in the EIDB and in the HIVdb TCE repository. This yielded two datasets of TCEs with binary labels for therapeutic success, the EuResistTCE (*n* = 1,650) and the HIVdbTCE (*n* = 1,000) datasets. Fig 2a) depicts the most frequent therapies in the EuResistTCE, while Fig 2b) does so for the TCEs in HIVdbTCE. The baseline sequences in EuResistTCE overlap with the sequences in PRRT partially; the baseline sequences of 619 TCEs are not included in PRRT. TCEs in EuResistTCE whose baseline sequences were obtained during a therapy pause were assigned to the EuResistTCE_TP_ test set. EuResistTCE contains 313 first-line therapies (19.0%) among which 44 (14.1%) present TDR in their baseline sequences. No therapy in HIVdbTCE is a first-line therapy. The Pheno dataset contains GPPs which were labeled *susceptible* or *resistant* using the resistance-factor (RF) cutoffs one and ten. Pheno was randomly split into the development and training sets D_Pheno_ and T_Pheno_, respectively (Methods). The compositions of D_Pheno_ and T_Pheno_ are displayed in Table 4.

**Table 2:** Number of nucleotide sequences by subtype and dataset.

Subtype	PRRT	IN	HIVdbExposure
B	42,634 (61%)	2,721 (49%)	1,377 (1%)
C	6,243 (9%)	1,293 (23%)	1 (< 1%)
A1	3,704 (5%)	166 (3%)	1 (< 1%)
G	3,223 (5%)	270 (5%)	0 (0%)
02_AG	3,010 (4%)	66 (1%)	1 (< 1%)
01_AE	4,275 (6%)	596 (11%)	1 (< 1%)
D	1,169 (2%)	53 (1%)	1 (< 1%)
F1	971 (1%)	69 (1%)	0 (0%)
06_cpx	312 (< 1%)	89 (2%)	0 (0%)
07_BC	651 (1%)	4 (< 1%)	0 (0%)
Other	4,112 (6%)	196 (4%)	2 (< 1%)
Total	70,304	5,523	1,381

Nucleotide sequences in the PRRT, IN, and HIVdbExposure datasets were subtyped with the Comet subtyping tool. Sequence counts for the ten most frequent subtypes are tabulated above. For each dataset, the percentage of nucleotide sequences with a particular subtype are stated in parenthesis.

**Table 3:** Number of sequences by dataset and drug exposure.

	D_PRRT_	D_PRRT_ Comp.	D_IN_	D_IN_ Comp.	T_PRRT_	T_PRRT_ Comp.	T_IN_	T_IN_ Comp.	TP	TP Comp.	HIVdbExposure
**ABC**	7,482	4,560	295	229	1,839	1,028	164	103	163	30	301
**AZT**	18,542	12,184	441	336	3,895	2,405	222	135	372	68	1,075
**d4T**	13,335	8,079	259	197	2,956	1,764	141	80	250	31	998
**ddC**	4,007	2,341	57	45	1,114	750	52	40	71	6	297
**ddI**	12,113	7,398	227	173	2,725	1,657	123	73	197	23	722
**FTC**	4,580	3,258	359	266	900	595	162	112	52	8	59
**3TC**	20,730	13,416	525	394	4,191	2,543	262	151	390	70	0
**TDF**	9,546	6058	479	356	1,933	1,192	211	130	119	13	219
**DLV**	118	56	5	2	96	58	26	18	6	1	73
**EFV**	9,673	6,228	301	238	2,168	1,310	168	110	194	35	400
**ETR**	255	169	62	51	145	94	70	48	4	3	1
**NVP**	8,405	5,044	232	178	1,836	1,054	123	74	179	31	508
**RPV**	5	4	2	1	0	0	0	0	0	0	0
**APV**	1,240	615	48	37	463	216	41	31	38	3	192
**ATV**	3,444	2,293	230	166	833	510	131	79	39	6	52
**DRV**	916	587	152	95	328	200	111	68	16	6	4
**FPV**	1,028	621	79	58	381	211	52	26	28	5	20
**IDV**	9,466	5,965	184	150	2,134	1,433	112	84	144	20	737
**LPV**	8,516	5,293	332	244	2,156	1,315	180	104	142	22	147
**NFV**	7,540	4,669	137	104	1,698	1,018	101	68	113	17	706
**SQV**	6,187	3,646	166	125	1,638	951	91	41	136	21	428
**TPV**	643	345	71	58	246	153	62	39	9	0	5
**EVG**	10	1	3	2	0	0	0	0	0	0	0
**RAL**	650	448	223	171	251	156	116	80	7	3	0
**Naïve**	37,577	37,577	1,917	1,917	2,056	2,056	154	154	3	3	0
**Total**	61,163	53,098	2,579	2,408	6,641	4,862	444	326	441	84	1,384

The numbers of sequences by drug exposure for the development and test datasets are tabulated above. Columns including the abbreviation *Comp*. in their headers indicate the numbers of sequences from a certain dataset and with a certain drug exposure whose complete drug exposure history is known. The complete drug exposure history for all sequences from the HIVdbExposure dataset is available.

3TC: lamivudine, ABC: abacavir, AZT: zidovudine, d4T: stavudine, ddC: zalcitabine, ddI: didanosine, FTC: emtricitabine, TDF: tenofovir, DLV: delavirdine, EFV: efavirenz, ETR: etravirine, NVP: nevirapine, RPV: rilpivirine, APV: amprenavir, ATV: atazanavir, DRV: darunavir, FPV: fosamprenavir, IDV: indinavir, LPV: lopinavir, NFV: nelfinavir, SQV: saquinavir, TPV: tipranavir, EVG: elvitegravir, RAL: raltegravir

**Table 4:** Number of phenotypes by drug in the pheno datasets.

	Antivirogram	PhenoSense	Susceptible	Resistant	Total
	**D**_**Pheno**_				
**3TC**	905	1546	346	1362	2451
**ABC**	840	1473	531	186	2313
**AZT**	855	1567	801	773	2422
**d4T**	889	1573	1031	60	2462
**ddC**	821	451	371	47	1272
**ddI**	891	1575	654	59	2466
**TDF**	633	1234	850	33	1867
**DLV**	1016	1638	794	1091	2654
**EFV**	1106	1652	924	1127	2758
**ETR**	363	476	304	156	839
**NVP**	1170	1653	772	1447	2823
**RPV**	91	176	62	75	267
**ATV**	774	1134	401	978	1908
**DRV**	282	629	400	178	911
**FPV**	1088	1695	917	859	2783
**IDV**	1151	1734	782	1229	2885
**LPV**	1040	1468	665	1279	2508
**NFV**	1185	1780	483	1584	2965
**SQV**	1181	1741	985	1039	2922
**TPV**	742	854	584	191	1596
**EVG**	97	598	112	137	695
**RAL**	97	630	336	148	727
	**T**_**Pheno**_				
**3TC**	115	166	37	158	281
**ABC**	107	166	60	25	273
**AZT**	107	165	92	88	272
**d4T**	110	168	122	6	278
**ddC**	105	46	38	5	151
**ddI**	111	168	72	7	279
**TDF**	87	132	87	4	219
**DLV**	126	169	81	125	295
**EFV**	141	171	105	136	312
**ETR**	43	52	36	15	95
**NVP**	146	175	82	170	321
**RPV**	14	21	13	10	35
**ATV**	85	131	42	115	216
**DRV**	22	79	50	20	101
**FPV**	110	193	88	105	303
**IDV**	125	194	76	142	319
**LPV**	113	172	76	151	285
**NFV**	127	199	48	189	326
**SQV**	129	195	105	119	324
**TPV**	80	106	56	22	186
**EVG**	17	61	9	11	78
**RAL**	17	65	36	21	82

The numbers of phenotypes by drug in the D_Pheno_ and T_Pheno_ datasets are tabulated above. Phenotypes were measured with the Antivirogram^™^ or PhenoSense^™^ assays. Resistance-factor cutoffs one and ten were used for dichotomizing phenotypes into *susceptible* and *resistant*.

3TC: lamivudine, ABC: abacavir, AZT: zidovudine, d4T: stavudine, ddC: zalcitabine, ddI: didanosine, FTC: emtricitabine, TDF: tenofovir, DLV: delavirdine, EFV: efavirenz, ETR: etravirine, NVP: nevirapine, RPV: rilpivirine, APV: amprenavir, ATV: atazanavir, DRV: darunavir, FPV: fosamprenavir, IDV: indinavir, LPV: lopinavir, NFV: nelfinavir, SQV: saquinavir, TPV: tipranavir, EVG: elvitegravir, RAL: raltegravir

### 2.2. Training of models for predicting drug exposure

We trained linear Support Vector classifiers (SVC) [47,48] for discriminating between sequences from viruses with and without previous exposure to a certain drug. We trained SVCs on two kinds of development sets, Exposure or ExposurePheno. Specifically, each sequence in the development sets D_PRRT_ and D_IN_ included binary labels indicating whether exposure to a particular drug had occurred or not. We used D_PRRT_ and D_IN_ for creating one Exposure_*drug*_ development set for each drug and subsequently trained one SVC on each of these development sets. We additionally created the development set Exposure_naïvePRRT_ in which labels indicate whether viral sequences were derived from therapy-naïve or therapy-experienced patients. Subsequently, we trained one SVC on Exposure_naïvePRRT_. For creating the ExposurePheno_*drug*_ development sets, we extended the data in the Exposure_*drug*_ development sets with GPPs from D_Pheno_. In the ExposurePheno_*drug*_ development sets, viral sequences from GPPs labeled as *susceptible* to the drug in question are treated as not having being exposed to the drug. Conversely, GPPs labeled as *resistant* to the drug in question are treated as having been exposed to the drug. We trained an SVC with each ExposurePheno_*drug*_ development set. We do not consider the binary output of the SVC classifier but rather the reported signed distance from the decision boundary, a real number. We call this number *drug-exposure score* (DES).

### 2.3. Assessment and comparison of performance

We constructed DES models with 10-fold cross validation on the respective development sets. Then we used the DES reported by the resulting models and the predicted resistance factor for geno2pheno_[resistance]_, respectively to calculate and compare AUC performance of both models. This was done for the test sets T_PRRT_, T_IN_, TP, HIVdbExposure, T_Pheno_, EuResistTCE, EuResistTCE_TP_, and HIVdbTCE. Among the 7,275 protease and reverse-transcriptase nucleotide sequences contained in T_PRRT_ and EuResistTCE, 23 (<0.01%) were not processed by geno2pheno_[resistance]_ due to low sequence similarity. For the sake of performance comparison, these sequences were excluded. In the following, mean performances for the tested models are stated. In order to be able to compare the different models, these means were calculated only with the performances of the drugs that are common to Exposure and ExposurePheno models, as well as to geno2pheno_[resistance]_. p-values were calculated with a two-sided Wilcoxon signed-rank test [49].

#### 2.3.1. Assessment of performance for predicting drug exposure via cross validation

**Fig 3:**
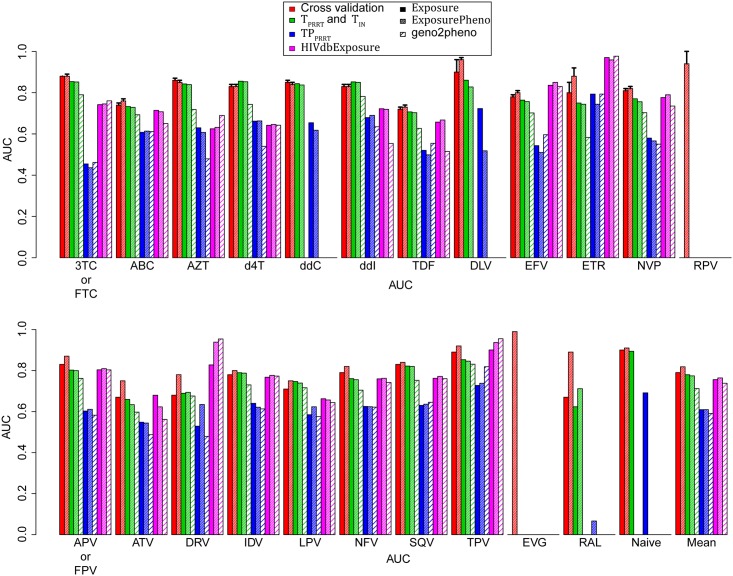
Performance of drug-exposure prediction. Performance of drug-exposure prediction was assessed with 10-fold cross validation on the development set and four test sets. Test sets T_PRRT_ and T_IN_ were obtained from the EuResist database and contain protease and reverse-transcriptase and integrase sequences, respectively. TP is a subset of T_PRRT_ ∪ T_IN_ and contains nucleotide sequences that were measured during therapy pauses. HIVdbExposure was obtained from the HIVdb TCE repository and contains protease and reverse-transcriptase sequences. Performance on the test sets was compared to that of geno2pheno_[resistance]_. Bars depicting mean performances were calculated only using drugs that are common to Exposure and ExposurePheno models, as well as to geno2pheno_[resistance]_. Error bars indicate the standard deviation. 3TC: lamivudine, ABC: abacavir, AZT: zidovudine, d4T: stavudine, ddC: zalcitabine, ddI: didanosine, FTC: emtricitabine, TDF: tenofovir, DLV: delavirdine, EFV: efavirenz, ETR: etravirine, NVP: nevirapine, RPV: rilpivirine, APV: amprenavir, ATV: atazanavir, DRV: darunavir, FPV: fosamprenavir, IDV: indinavir, LPV: lopinavir, NFV: nelfinavir, SQV: saquinavir, TPV: tipranavir, EVG: elvitegravir, RAL: raltegravir.

We cross validated the SVC on each Exposure_*drug*_ and each ExposurePheno_*drug*_ development set, as well as on the Exposure_naïvePRRT_ development set. Specifically, we performed ten repetitions of a five-fold cross validation on each development set while testing a series of values for the SVC *c* parameter (see Methods). One value of the *c* parameter was chosen for each development set. The mean drug-wise cross-validation performances (area under the receiver operating characteristic curve; AUC) for the chosen values of *c* ranged between 0.67 and 0.99. Models trained on ExposurePheno cross-validation sets had a higher mean cross-validation performance (*μ* = 0.82; *σ* = 0.05) than those trained on Exposure cross-validation sets (*μ* = 0.79; *σ* = 0.07; p < 0.003). The p-value quantifies the difference in the AUC distributions between Exposure and ExposurePheno models. Individual performances are depicted in Fig 3.

#### 2.3.2. Assessment of performance for predicting drug exposure on test sets

The performances of DES for predicting drug exposure on the T_PRRT_, T_IN_, TP, and HIVdbExposure test sets are depicted in Fig 3. The performance of geno2pheno_[resistance]_ on T_PRRT_, TP, and HIVdbExposure sets can be seen in Fig 3 as well. In the following, p-values quantify the difference in the AUC distributions between Exposure models, ExposurePheno models or geno2pheno_[resistance]_. The best mean performance on the T_PRRT_ dataset could be attained by Exposure models (*μ* = 0.78; *σ* = 0.06), while the mean performance of geno2pheno_[resistance]_ was lower (*μ* = 0.71; *σ* = 0.07; p < 10^−4^). On the T_IN_ dataset, DES performance for RAL was comparable for models trained on ExposurePheno cross-validation sets (AUC = 0.71), but not for those trained on Exposure cross-validation sets (AUC = 0.62). On the HIVdbExposure dataset, the best mean performance with lowest standard deviation was achieved with Exposure models (*μ* = 0.76; *σ* = 0.09), while geno2pheno_[resistance]_ achieved a lower mean performance (*μ* = 0.74; *σ* = 0.14; p = 0.43). The best mean performance on TP could be attained with Exposure and ExposurePheno models (*μ* = 0.61; *σ* = 0.08), while geno2pheno_[resistance]_ displayed a lower mean performance (*μ* = 0.59; *σ* = 0.10; p = 0.3778).

#### 2.3.3. Assessment of performance for predicting drug resistance

**Fig 4:**
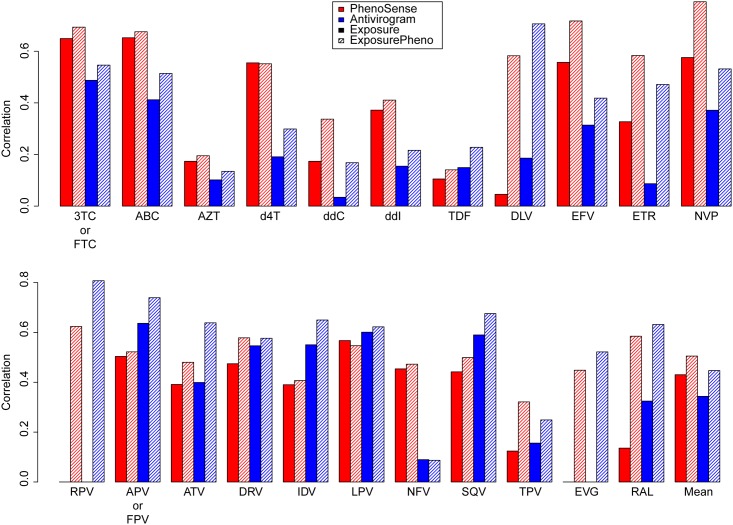
Correlation of drug-exposure scores with logarithmized resistance factors. Genotypes in T_Pheno_ were interpreted with drug-exposure models. The correlation of the resulting drug-exposure scores with the corresponding logarithmized resistance factors is displayed above. Note that drug-resistance assays (either Antivirogram^™^ or PhenoSense^™^) are denoted by the colors of the bars, while the drug-exposure model types (Exposure or ExposurePheno) are denoted by the shading of the bars. Bars depicting the mean performances were calculated with the drugs for which Exposure and ExposurePheno models are available. Error bars indicate the standard deviation. 3TC: lamivudine, ABC: abacavir, AZT: zidovudine, d4T: stavudine, ddC: zalcitabine, ddI: didanosine, FTC: emtricitabine, TDF: tenofovir, DLV: delavirdine, EFV: efavirenz, ETR: etravirine, NVP: nevirapine, RPV: rilpivirine, APV: amprenavir, ATV: atazanavir, DRV: darunavir, FPV: fosamprenavir, IDV: indinavir, LPV: lopinavir, NFV: nelfinavir, SQV: saquinavir, TPV: tipranavir, EVG: elvitegravir, RAL: raltegravir.

Fig 4 shows the correlation of DES with the logarithmized resistance factors from the T_Pheno_ dataset. DES models trained on ExposurePheno cross-validation sets could attain substantially higher mean correlations (*μ*_Antivirogram_ = 0.46; *μ*_PhenoSense_ = 0.51; *σ*_Antivirogram_ = 0.2; *σ*_PhenoSense_ = 0.17) than those trained on Exposure cross-validation sets (*μ*_Antivirogram_ = 0.34; *μ*_PhenoSense_ = 0.41; *σ*_Antivirogram_ = 0.2; *σ*_PhenoSense_ = 0.18). We consider this sufficient correlation for the intended use of our software. Furthermore, it should be noted that the between-test correlation of PhenoSense and Antivirogram is weak (r = 0.36) [11].

#### 2.3.4. Assessment of performance for predicting therapy success

We tested the performance of DES models and of geno2pheno_[resistance]_ in predicting the binary therapy-success labels of the TCEs in EuResistTCE, EuResistTCE_TP_, and HIVdbTCE. For this purpose, we used DES and geno2pheno_[resistance]_, respectively, for calculating a genetic susceptibility score (GSS) for each TCE, an additive score that rates the susceptibility of the virus to the used drugs. For GSS calculation, the predictions of DES models and of geno2pheno_[resistance]_ were translated into probability scores (Methods*)*. The GSS for a TCE is the sum of the probability scores for its constituent drug compounds. The performances of GSS calculated with Exposure models, ExposurePheno models, and geno2pheno_[resistance]_, respectively, when predicting binary labels for therapeutic success are displayed in Table 5. On the EuResistTCE dataset, the best performance could be attained with Exposure and ExposurePheno models (AUC = 0.71), while the performance of geno2pheno_[resistance]_ was lower (AUC = 0.68). On therapies with baseline sequences measured during a therapy pause (EuResistTCE_TP_), ExposurePheno models displayed the best performance (AUC = 0.73), while the performance of geno2pheno_[resistance]_ was lower (AUC = 0.66). The best performance on HIVdbTCE is displayed by geno2pheno_[resistance]_ (AUC = 0.64), while the performance of drug-exposure models was lower (AUC ≤ 0.63). On average, ExposurePheno models displayed the highest performance with lowest standard deviation in predicting therapeutic success (*μ* = 0.69; *σ* = 0.05). The average performance of geno2pheno_[resistance]_ when predicting therapeutic success was lower (*μ* = 0.66; *σ* = 0.02; p = 0.5).

**Table 5:** Performance of prediction of therapy-success for therapies in EuResistTCE, EuResistTCE_TP_, and HIVdbTCE.

	Exposure	ExposurePheno	geno2pheno_[resistance]_
**EuResistTCE**	0.71	0.71	0.68
**EuResistTCE**_**TP**_	0.72	0.73	0.66
**HIVdbTCE**	0.62	0.63	0.64

Therapy success was predicted for therapies in the EuResistTCE, EuResistTCE_TP_, and HIVdbTCE test using three different genetic susceptibility scores (GSS) for each therapy. The first GSS was obtained with Exposure models, the second GSS with ExposurePheno models and the third GSS with geno2pheno_[resistance]_. Above, the performances of the three different GSS are tabulated for each dataset. Performances were quantified with the area under the receiver operating characteristic curve.

### 2.4. Assessment of performance of drug-exposure models with cutoff-based categorization

HIV-1 nucleotide sequences can be submitted to our web service for interpretation with ExposurePheno models (see Discussion). For the purpose of facilitating the use of DES by human experts, we estimated cutoffs for translating DES into clinically meaningful categories. We estimated two sets of cutoffs (see Methods for details). DEMax cutoffs translate DES into categories describing degrees of drug exposure (Table A in S1 File), while pheno cutoffs translate DES into categories describing degrees of drug resistance (Table B in S1 File). For determining and testing pheno cutoffs, we applied clinically relevant RF cutoffs to PhenoSense GPPs in Pheno (Table C in S1 File). After discretization of DES with DEMax cutoffs, we calculated their performance when predicting drug exposure on T_PRRT_, T_IN_, TP, and HIVdbExposure in terms of AUC (Table D in S1 File). Furthermore, we discretized DES with pheno cutoffs and calculated their misclassification rates when predicting drug resistance in T_Pheno_ (Table E in S1 File). The application of cutoffs to DES can be associated with a mild loss in predictive performance. When predicting drug exposure without application of cutoffs, average performances (AUC; mean calculated with all drugs for which an ExposurePheno model is available) on the T_PRRT_ and T_IN_, TP, and HIVdbExposure datasets are 0.77 (0.06), and 0.57 (0.1), and 0.76 (0.11), respectively. After application of cutoffs, mean performance in predicting drug exposure is 0.76 (0.06), 0.58 (0.14), and 0.76 (0.11), respectively. When predicting phenotypic drug resistance, performance with and without application of cutoffs is difficult to compare since different performance measures are required for each case. Specifically, without application of cutoffs, performance was measured with the Pearson correlation coefficient, while with application of cutoffs, performance was measured with the misclassification rate. With application of cutoffs, the mean misclassification rate is 0.27 (0.1) for all GPPs, 0.11 (0.06) for *susceptible*-labeled GPPs, 0.1 (0.06) for *intermediate*-labeled GPPs, and 0.05 (0.03) for *resistant*-labeled GPPs.

## 3. Discussion

DES models constitute data-driven interpretation systems for HIV-1 protease, reverse-transcriptase, and integrase sequences. Two versions of DES models were trained and tested. Specifically, one version of the models is solely trained on genotypes and drug exposure information (Exposure models), while the other version additionally includes GPPs (ExposurePheno models). When compared to ExposurePheno models, Exposure models show a high performance when predicting drug exposure, but their correlation with RFs and their performance when predicting antiretroviral therapy success are lower. We chose to include GPPs in the training sets of ExposurePheno models for the following reasons. Both drug exposure and drug resistance are predictive of success of antiretroviral therapy [34,38–40]. The major factor leading to viral drug resistance is exposure to antiretroviral drugs. Specifically, drug resistance arises through the selection of HIV-1 strains with mutations that confer a replicative advantage in the presence of the drug. Thus, drug exposure indirectly causes drug resistance and therefore, both drug exposure and drug resistance are correlated with certain mutations in the genome of HIV-1. Nevertheless, drug exposure and drug resistance are not redundant, but can complement each other. For this reason, simultaneous interpretation of HIV-1 genotypes with respect to drug exposure and to drug resistance is useful for the prediction of the success of antiretroviral therapy. ExposurePheno models consider drug exposure and drug resistance jointly. For the purpose of including GPPs in the training set of classification models, RFs required categorization. Thus, we replaced the RFs in the GPPs with the labels *susceptible* and *resistant*. For the purpose of labeling, the RF cutoffs one and ten were applied to all GPPs, regardless of the drug-resistance test (Antivirogram or PhenoSense) and of the tested drug. GPPs with RFs between one and ten were not used for training the models. When clinically relevant categorization of GPPs is intended, different cutoffs for each drug and drug resistance test must be used [15]. However, rather than producing clinically relevant labels for training, we aimed at discriminating fully susceptible GPPs from those that have developed resistance to an extent well beyond the variability arising from the drug resistance test itself, for the following reasons. First, drug resistance is a continuum, and the creation of training instances with a clear separation in this continuum is adequate for the training of binary classification models. Second, clinically relevant cutoffs are selected under the (implicit) consideration of the pharmacokinetic properties of a drug. For example, the use of ritonavir as a booster for protease inhibitors (PIs) leads to an increased and sustained concentration of PIs in the body [50]. For this reason, clinically relevant cutoffs for boosted PIs are shifted upwards with respect to their unboosted counterparts [15]. However, we aim at discriminating viral sequences that display mutations as a consequence of drug exposure (or as the cause of resistance), without regard for drug concentrations in the blood of patient. The cutoffs one and ten are adequate for combining GPPs produced with the Antivirogram and PhenoSense assays; if other assays are used, other cutoffs might need to be selected. One advantage of ExposurePheno models over Exposure models is their higher performance. Another advantage is that they can make use of an additional data source, the GPPs. The use of GPPs allowed for the training of models for two additional drugs (EVG and RPV).

The interpretations provided by DES models can be used to addresses three questions: (1) Was an HIV-1 variant exposed to a certain drug? (2) Is an HIV-1 variant resistant to a certain drug? and (3) How does the effect of a drug combination therapy decompose into effects of its constituent drugs? In the following, we propose how DES models can be used for addressing the three questions mentioned above.

Ad question (1): When the prediction of drug exposure is required, we propose two ways in which DES can be used. For quantification and comparison of the degree of drug exposure between at least two groups of patients, we do not recommend translating DES into categories (by using cutoffs), since this leads to loss of precision. Instead, DES should be directly used for detecting differences between groups. Note that comparison of DES for different drugs requires normalization, e.g. via the calculation of z-scores (this is provided by our web service). If the prediction of the drug exposures of individual patients is required, cutoffs can be used in order to translate DES into clinically meaningful categories (this is also provided by our web service).

Ad question (2): When predicting drug resistance with DES, one should bear in mind that the correlation of DES with log RFs is weak to strong, depending on the drug in question (Fig 4). Correlation with drug resistance to the nucleotide and nucleoside reverse-transcriptase inhibitors (NRTIs) AZT, d4T, ddC, ddI, and TDF is lower than with resistance to other drugs. We interpret this to be the result of the high similarity of the resistance profiles among these NRTIs [17]. Mutations conferring resistance to one of these NRTIs confer resistance to the other NRTIs (cross-resistance) and are thus less discriminative of exposure and resistance to any specific drug among the NRTIs we mention above. Nonetheless, the correlation is sufficient for predicting the susceptible-intermediate-resistant (SIR) label of GPPs discretized with clinically relevant cutoffs (Table E in S1 File). While a mean misclassification rate of 0.27 (0.1) seems high, most of errors arise from misclassifying *intermediate*-labeled GPPs (*μ* = 0.1; *σ* = 0.06), for which the clinical relevance is uncertain [12], and from misclassification of *susceptible*-labeled GPPs that are predicted to be *intermediate* or *resistant* (*μ* = 0.1; *σ* = 0.06). For the drugs AZT, d4T, ddI, and TDF, misclassification of *susceptible*- and *intermediate*-labeled GPPs as *resistant* is especially high, which we also attribute to the high similarity of their resistance profiles. Misclassifying *susceptible*-labeled GPPs as *intermediate* or *resistant* can be clinically adequate, for the following reason. Phenotypic resistance measurements do not account for mutations that do not cause drug resistance *at the time of resistance testing*, but are indicative that drug resistance can be easily developed in the future [16]. Therefore, when such resistance mutations are present in the viral baseline genotype of a patient, and even if phenotypic resistance measurements indicate full drug susceptibility, classification of the genotype as non-susceptible will prevent selecting a combination of drug compounds that could quickly fail due to the emergence of drug resistant viral variants. Nonetheless, misclassifying *susceptible*-labeled GPPs as *intermediate* or *resistant* could also lead to rejection of a drug for treating a patient although the drug could have been a good choice. When the prediction of the results of phenotypic resistance tests is required, we recommend the use of interpretation systems that have been specifically designed and validated for this purpose, e.g. geno2pheno_[resistance]_. DES predictions are especially useful in two situations: first, when (imminent) drug resistance is not detected by other methods because the process of selection of drug-resistant variants has led to the selection of certain mutations that have not (yet) resulted in clinically relevant drug-resistance (Fig 3; ExposurePheno models have a higher performance in predicting drug exposure than geno2pheno_[resistance]_). Second, when drug-resistant HIV-1 variants are in the process of reverting to the *wild type* after the removal of drug pressure (Fig 3 and Table 5; ExposurePheno models have a higher performance in predicting drug exposure and therapeutic success than geno2pheno_[resistance]_ when genotypes were obtained during therapy pauses). Decision support for the choice of the use of the tool will be given in our follow-up paper.

Ad question (3): DES are predictive of therapeutic success (Table 5). In order to facilitate the use of DES for deciding which drug could be useful components of combination antiretroviral therapy, as well as for interpretation of DES by human experts, our online web service offers the following features. (i) Calculation of DES. (ii) Calculation of z-scores, which normalize DES with respect to their distribution in therapy patients. These z-scores can be useful when DES for different drugs need to be compared or merged for the analysis of clinical data. (iii) Translation of DES into categories related to drug exposure and drug resistance via cutoffs. HIV-1 can mutate as a result of exposure to antiretroviral drugs, which does not necessarily entail clinically relevant drug resistance. Drug-exposure categories help the user to determine whether a viral variant has changed as a result of drug exposure. If a viral variant is not rated *unexposed* for a certain drug, drug resistance to that drug should be at least suspected. Drug-resistance categories indicate whether viral mutations are not only indicative of exposure to a particular drug, but also indicative of clinically relevant drug resistance. If a viral variant is not rated *susceptible*, drug resistance is highly likely. Drug-exposure and drug-resistance categories are useful when selecting the drug components of antiretroviral therapy. However, predictions are provided for each drug individually (as in most drug-resistance interpretation tools). Thus, the selection of an adequate drug combination under consideration of DES is still left to the expert. In a follow-up paper we will report on a DES-based model that does not require expert selection of drug combinations. Specifically, we are currently testing DES as input features for a model for predicting the success of combinations of antiretroviral drugs. This model will exploit DES for selecting the compounds of antiretroviral therapy. (iv) Presentation of the basis of the predictions by displaying the residues with the largest influence on the prediction.

In summary, in this study, we present a novel approach for training a data-driven interpretation system for drug exposure and drug resistance. We show that models trained on HIV-1 sequences from patients with known drug history can be used for predicting drug exposure, drug resistance, and therapeutic success, even if no GPPs are used. The inclusion of GPPs in the training sets of the models boosted the performance of the models when predicting *in-vitro* phenotypic drug resistance measurements and therapeutic success, but not when predicting drug exposure. Compared to geno2pheno_[resistance]_, the method could attain higher mean performances when predicting drug exposure and therapeutic success. The difference in performance was only statistically significant at the 5% level when predicting drug exposure on T_PRRT_. Note that many of the drugs in HIVdbTCE are not used any more due to their toxicity profiles or their comparatively low potency. A large advantage of DES models is that they are trained on clinical HIV data and freely available GPPs. In conjunction with a frequently updated database with HIV-1 data from routine clinical practice, such as the EIDB, DES models can be automatically updated on a regular basis. Thus, these models allow us to reduce our dependency on hard-to-obtain GPPs for offering a publicly available data-driven genotypic drug-exposure and drug-resistance interpretation system that is kept up to date. While regularly updatable interpretation systems are clearly the appropriate method for accounting for the growing richness of clinical data, innovative procedures may have to be put in place for adequate certification of such systems. DES models for protease and reverse transcriptase inhibitors have been integrated into the geno2pheno_[resistance]_ server http://www.geno2pheno.org. Support for integrase inhibitors will follow. After a sequence has been submitted for prediction, the tab labeled *Drug Exposure* must be selected in order to view DES predictions. Note that in the input tab, sample nucleotide sequences can be loaded by selecting the appropriate action. On the website, mutations with the highest influence on the prediction are displayed. These are ordered by the magnitude of their influence. Mutations colored in red increase DES, while those colored in green decrease it.

## 4. Materials and methods

### 4.1. Ethics statement

All data considered in this study had been previously de-identified. For this reason, consent was neither required nor given by human subjects.

### 4.2. Drugs considered in this study

In this study, the following antiretroviral drugs are considered: lamivudine (3TC), abacavir (ABC), zidovudine (AZT), stavudine (d4T), zalcitabine (ddC), didanosine (ddI), emtricitabine (FTC), tenofovir (TDF), delavirdine (DLV), efavirenz (EFV), etravirine (ETR), nevirapine (NVP), rilpivirine (RPV), amprenavir (APV), atazanavir (ATV), darunavir (DRV), fosamprenavir (FPV), indinavir (IDV), lopinavir (LPV), nelfinavir (NFV), saquinavir (SQV), tipranavir (TPV), raltegravir (RAL) and elvitegravir (EVG). Other antiretroviral drugs were excluded due to insufficient data.

### 4.3. Dataset construction

In the following, we describe a number of datasets that we used for training and validating our models. For the comfort of the reader, we summarize the contents of each of these datasets in Table 1. Furthermore, we depict the relationships of each of the datasets in Figure A in S1 File.

#### 4.3.1. Datasets of genotypes and therapeutic history

The PRRT and IN datasets contain HIV-1 nucleotide sequences and information on the antiretroviral compounds that were used before each sequence was obtained. When constructing these datasets, we disregarded episodes of treatment with a drug that lasted less than 30 days. The PRRT dataset was constructed by pooling 70,304 HIV-1 protease (PR) and reverse-transcriptase (RT) nucleotide sequences from two sources: 37,799 sequences from the EuResist Integrated Database (EIDB; http://www.euresist.org, downloaded April 11^th^, 2014) [45], 9,627 of which were derived from drug-naïve patients (short: drug-naïve sequences), and 32,506 drug-naïve sequences from the Los Alamos National Laboratory Sequence Database (LANLSD; http://www.hiv.lanl.gov/; downloaded on March 31^st^, 2015). Among the sequences in the PRRT dataset that were derived from therapy-experienced patients (short: drug-exposed sequences), 18,328 sequences were derived from patients whose complete drug history was available at the time of sequencing. The IN dataset includes a total of 5,523 integrase (IN) nucleotide sequences with the following characteristics: 3,382 sequences were extracted from the EuResist database, 1,240 of which are drug-naïve, 397 have been exposed to an integrase inhibitor (INI) and possibly other drugs, and 1,745 have been exposed only to drugs whose target is different from integrase. The complete drug history was available for 1,432 of the drug-exposed integrase sequences. Additionally, 3,782 drug-naïve integrase sequences from the LANLSD (downloaded on March 31^st^, 2015) were added to the IN dataset. Inclusion criteria for the sequences were as follows. (1) Alignment with the *MutExt* software (http://www.schuelter-gm.de/mutext.html) must not have produced an error due to low sequence similarity to the reference sequence (2) at most 10% of the residues of the considered protein regions could not be determined by the sequencing procedure (considered protein regions are listed in Section *Subtype Determination*, *Sequence Alignment and Encoding*), (3) the amino-acid sequence resulting from nucleotide translation must be unique within the dataset, unless drug exposure differed between duplicates. The order of appearance of the sequences in the dataset determined which duplicate sequence was excluded, with sequences appearing first preempting inclusion of sequences appearing later. Older reverse-transcriptase sequences not covering amino-acid positions 221–230 were excluded as well.

PRRT and IN were split into development and test sets, as follows. For the purpose of rigorous validation, HIV-1 nucleotide sequences derived from the same patient were not allowed to be simultaneously present in the development and test sets. In the following, dataset nomenclature consists of an abbreviation describing a characteristic of the dataset and optionally PRRT or IN in subscript. The letters in subscript indicate whether the dataset is a subset of the PRRT or of the IN dataset. All HIV-1 nucleotide sequences in PRRT and IN that were obtained during a therapy pause were assigned to TP (*n* = 441). This dataset is only used for testing purposes, since we deem therapy-pause sequences valuable for testing and an insignificant minority in the much larger training set. In order to make sure that the test sets are patient-wise disjoint with respect to the development set, a set of test patients P was created iteratively. Initially, P included all patients with sequences in TP. Further patients from PRRT and IN were subsequently added to P by random selection until the number of available sequences for the patients in P was approximately 10% of the number of sequences in PRRT and IN. The test sets T_PRRT_ and T_IN_ contain the protease and reverse-transcriptase sequences available for the patients in P, respectively. The development sets D_PRRT_ and D_IN_ contain the sequences in PRRT and IN, respectively, that are not included in T_PRRT_, and T_IN_.

#### 4.3.2. EuResistTCE dataset and standard datum definition

In order to test the performance of our models in predicting therapeutic success, we created the EuResistTCE test set, as follows. We extracted a total of 9,201 therapy-change episodes (TCEs) from the EIDB [45]. These TCEs were constructed according to the definition of the EuResist Standard Datum [40]. In contrast to the EuResist Standard Datum, however, viral-load (VL) measurements were constrained to those not reaching a lower limit of quantification greater or equal than 50 HIV-1 RNA copies per milliliter of blood plasma. In summary, each TCE includes a protease and reverse transcriptase baseline sequence, the compounds that were prescribed to the patient, a baseline and a follow-up viral load (VL), and a binary label indicating whether the therapy was successful or not. The follow-up VL must have been measured within 4–12 weeks after therapy start, preferring the VL closest to week 8 after therapy start. Therapy success at follow up is defined as an at least 100-fold reduction in the VL or a VL of less than 400 HIV-1 RNA copies per ml of blood plasma. This definition of therapy success was used for producing binary labels for the TCEs. To allow performance comparison, only therapies including the following antiretroviral drugs were considered: 3TC, ABC, AZT, d4T, ddI, FTC, TDF, EFV, ETR, NVP, APV, ATV, DRV, FPV, IDV, LPV, NFV, SQV, TPV and ritonavir as a boosting agent (/r). Therapies including unboosted protease inhibitors (except for NFV, since the drug cannot be boosted) were excluded due to their comparatively inferior potency.

The baseline sequences of the EuResist TCEs partially overlap with the sequences in the datasets described above. A minority of baseline sequences were not included in any of the datasets described above because we could not ascertain whether drug exposure had occurred or the patient was therapy-naïve at the time of sequencing. We created a set of test TCEs, EuResistTCE, with a fraction of the initially extracted EuResist TCEs. Specifically, EuResistTCE only contained TCEs with baseline sequences that had not been derived from a patient with an HIV-1 nucleotide sequence in D_PRRT_ or in D_IN_. A subset of the TCEs in EuResistTCE includes baseline sequences which were obtained during a therapy pause. We refer to these TCEs as EuResistTCE_TP_.

#### 4.3.3. HIVdbExposure and HIVdbTCE datasets

For testing the performance of our models in predicting drug exposure and therapeutic success, we created the HIVdbExposure and the HIVdbTCE test sets, respectively. The TCE repository in the HIV Drug Resistance Database was downloaded in its entirety on November 21^st^, 2013 [14,46]. The TCE repository contains 58 TCEs from the EuResist database, which were discarded. A total of 1,384 sequences with drug-exposure information could be extracted from the repository. We assigned these sequences to the HIVdbExposure test set. For creating the HIVdbTCE test set, the EuResist Standard Datum definition was applied to therapies in HIVdbTCE whose drug compounds are investigated in this study (with the exception of ddC and raltegravir (RAL) for the sake of performance comparison).

#### 4.3.4. Datasets of genotype-phenotype pairs

A total of 7,597 GPPs were downloaded from the HIV Drug Resistance Database [14] on April 15^th^, 2015 (Pheno dataset). The phenotypic drug-resistance assays used for producing the phenotypes were constrained to Antivirogram^®^ [51] and PhenoSense^®^[52]. The genotypes are provided in the form of substitutions with respect to the reference sequence *consensus B* [14]. 3,323 GPP quantify protease-inhibitor (PI) resistance, 3,477 reverse-transcriptase-inhibitor (RTI) resistance, and 797 INI resistance. The T_Pheno_ test set was created from the Pheno dataset by randomly sampling approximately 10% of the GPP. The rest of the GPPs in Pheno were assigned to the D_Pheno_ development set. For training our models with the GPPs, we categorized the resistance factors (RFs) in D_Pheno_ as *susceptible* or *resistant*. Specifically, GPPs with RFs lower or equal to one were categorized as *susceptible*, while GPPs with RFs greater or equal to ten were categorized as *resistant*. GPPs with RFs between one and ten were not used for training our models.

#### 4.3.5. Naïve_PRRT_ and Naïve_IN_ datasets

Transmitted drug resistance (TDR) in PR- and RT-naïve sequences was defined as the presence of at least one mutation in the list of drug resistance mutations for surveillance of transmitted HIV-1 drug resistance (DRMT) [7]. Since the list of DRMT only contains PR and RT mutations, TDR in IN sequences was defined as the presence of an INI drug-resistance mutation in the 2013 IAS list [17]. Following the methodology used for establishing the list of DRMT, INI drug-resistance mutations with a prevalence greater than 0.5% among sequences from the LANLSD in IN were not regarded as indicative of TDR [28]. The Naïve_PRRT_ and Naïve_IN_ were created by randomly sampling 2,500 LANLSD sequences without TDR from the PRRT and IN datasets, respectively. These sequences are not included in T_PRRT_, T_IN_, D_PRRT_ or D_IN_. Naïve_PRRT_ and Naïve_IN_ are used by our web service for z-score calculation.

### 4.4. Subtype determination, sequence alignment and encoding

The subtype distribution in the PRRT and IN datasets was determined with the COMET subtyping tool [53]. Nucleotide sequences in PRRT and IN were aligned against HXB2 and translated, using MutExt (http://www.schuelter-gm.de). The resulting amino-acid sequences, along with amino-acid sequences in the Pheno dataset, were represented vectorially with a binary encoding. The vectorial representation considers substitutions, deletions and the presence of insertions within the following HXB2 amino-acid positions: protease 3–99, reverse transcriptase 40–230, and integrase 30–260. The presence of deletions and insertions was encoded for each amino-acid position, while the amino acids of which a specific insertion consists were not encoded.

### 4.5. Creation of exposure and ExposurePheno development sets

D_PRRT_ and D_IN_ were used for constructing the development sets Exposure_*drug*_ for *drug* ∈ {ABC, AZT, d4T, ddC, ddI, 3TC/FTC, TDF, EFV, ETR, NVP, RPV, ATV, DRV, APV/FPV, IDV, LPV, NFV, SQV, TPV, RAL, EVG} which contain an equal number of sequences exposed and not exposed to a certain drug, along with binary labels indicating exposure to the drug. Sequences not exposed to the drug were randomly selected from D_PRRT_ or D_IN_, as they were in excess; these sequences were required to have been derived from patients whose complete drug exposure history is recorded. Where possible, half of the sequences not exposed to the drug were drug-naïve, and half of them were exposed to some other drug. A development set Exposure_naïvePRRT_ containing an equal number of drug-naïve and drug-experienced PRRT sequences was constructed as well. An Exposure_naïveIN_ development set was not created due to the fact that only a sufficient number of RAL-exposed integrase sequences was available.

The ExposurePheno_*drug*_ development sets were created from the Exposure_*drug*_ sets with additional supplementation of some GPPs from the D_Pheno_ dataset. Specifically, genotypes with corresponding RFs classified as resistant were treated as drug-exposed sequences while those with corresponding RFs classified as susceptible were treated as sequences not exposed to the drug in question. Genotypes with corresponding RFs between the two cutoffs were not used for training (see *Phenotypic Resistance Cutoffs*). This procedure incremented the number of available drug-exposed sequences and allowed for the creation of the development sets ExposurePheno_RPV_ and ExposurePheno_EVG_, as the number of available drug-exposed sequences for RPV and EVG was very low. Development sets for dolutegravir could not be created, as neither a sufficient number of resistant phenotypes nor a sufficient number of drug-exposed sequences were available.

### 4.6. Training and selection of models for predicting drug exposure

For performing five repetitions of a 10-fold cross validation, each Exposure_*drug*_ and each ExposurePheno_*drug*_ set was randomly partitioned five times into ten folds. Each fold contained an equal proportion of sequences with and without exposure to the drug in question. The partitions were used to cross validate linear support-vector classifiers (SVCs) [47,48] discriminating between sequences with and without exposure to a certain drug. The vectorial representation used to train each drug-specific model was constrained to the vector elements describing the drug’s target protein (protease, reverse transcriptase or integrase). Each cross validation was performed with a certain value for the regularization parameter *c* for the SVM, specifically, *c* ∈ {2^−8^, 2^−7^, …, 2^2^}. Performance was measured in terms of the area under the receiving-operator-characteristic curve (AUC) [54,55]. The signed distance to the classification hyperplane (also called decision value) was used as a score for predicting drug exposure. Thus, we call such decision values drug-exposure scores (DES). For each cross-validation set and vectorial representation, the model with the lowest value of *c* whose average performance was not significantly lower than the best average performance was selected (Benjamini-Hochberg-corrected Wilcoxon signed-rank test [49] with a significance threshold of 0.05). Finally, each cross-validation set and vectorial representation was used without partitioning to train a final SVC with the selected value of *c*. We refer to these SVCs as the final DES models.

### 4.7. Assessment and comparison of performance

The performance of the drug-exposure models was compared to that of geno2pheno_[resistance]_ 3.3 (http://www.geno2pheno.org) [20]. The output of geno2pheno_[resistance]_ includes a prediction of the resistance-factor (RF). Since geno2pheno_[resistance]_ uses its own alignment program, performance comparison was constrained to the set of sequences which could be aligned without errors by geno2pheno_[resistance]_. Furthermore, the drug ddC was also excluded from performance comparison, as it is not supported by geno2pheno_[resistance]_ any more.

#### Assessment of performance

Sequences in T_PRRT_, T_IN_, TP, EuResistTCE, EuResistTCE_TP_, HIVdbTCE, HIVdbExposure, and T_Pheno_ were interpreted with the final drug-exposure models and geno2pheno_[resistance]_. Performance was assessed in three respects. First, the performance of DES and of geno2pheno_[resistance]_ when predicting drug exposure was assessed using T_PRRT_, T_IN_, TP, and HIVdbExposure. These datasets contain HIV-1 sequences and a binary matrix indicating the previous exposure of these sequences to each individual drug compound. The area under the receiver operating characteristic curve (AUC) was used as a performance measure. Second, the performance of DES when predicting drug resistance was quantified with the correlation between DES for the genotypes in T_Pheno_ and the corresponding log RFs. Unfortunately, performance in predicting drug resistance could not be compared to that of geno2pheno_[resistance]_, since the genotypes in T_Pheno_ were only available as amino-acid sequences and geno2pheno_[resistance]_ requires nucleotide sequences as an input. Third, the performance of DES and of geno2pheno_[resistance]_ when predicting therapy success was assessed with EuResistTCE, EuResistTCE_TP_, and HIVdbTCE. For this purpose, DES and RF predictions were converted to probability scores. Specifically, DES, which are SVM decision values, were converted to probability scores as described by Platt [56]. Predicted RFs were converted to probability scores by fitting a two-component Gaussian-mixture model. In the Gaussian mixture model, one Gaussian is fitted to RFs that belong to the susceptible population, while the other Gaussian is fitted to RFs that belong to the resistant population. Subsequently, a sigmoid function is used for estimating the probability of resistance [20]. We define the probability of susceptibility as one minus the probability of resistance. Probability scores were used for calculating a genetic susceptibility score (GSS) for each therapy. A GSS consisted of the sum of the individual probability scores for each drug in the regimen. For each therapy, three GSS were calculated. The first two GSS were calculated using the probability scores derived with DES from Exposure and ExposurePheno models, respectively, while the third GSS was calculated with the probability scores derived with geno2pheno_[resistance]_. Performance in predicting therapeutic success was quantified with the AUC. Significance values in the *Results* section were calculated with a two-sided Wilcoxon signed-rank test [49].

### 4.8. Determination of parameters for our web service

Our drug-exposure models are freely available online (http://www.geno2pheno.org; see Discussion). For the purpose of facilitating the use of DES by human experts, we calculated two sets of parameters. The first set of parameters is used for calculating z-scores of DES. It includes a mean and a standard-deviation value for each drug, calculated with the nucleotide sequences in Naïve_PRRT_ and Naïve_IN_. The second set of parameters includes cutoffs which translate DES into clinically meaningful categories related to drug exposure and drug resistance. For the purpose of displaying the sequence features with the largest influence on the predictions of DES models, we translated the Support Vectors and corresponding Support-Vector coefficients of each SVC into a linear function. In the following, we detail on the procedures we used for determining the z-score parameters, the cutoffs, and for extracting the weights for the input features.

#### 4.8.1. Calculation of z-scores from drug-exposure scores

We interpreted each sequence in Naïve_PRRT_ with each DES model for predicting exposure to protease inhibitors (PIs) and reverse-transcriptase inhibitors (RTIs). Likewise, we interpreted each sequence in Naïve_IN_ with each DES model for predicting exposure to INIs. For each drug, we calculated the mean and standard deviation of the resulting DES. Our web service calculates the z-score for a sequence *s* and compound *drug* according to the following formula

$$z_{drug}\left(s\right)=\frac{\delta_{drug}\left(s\right)-\mu_{drug}}{\sigma_{drug}},$$ (1)

where *z*_*drug*_(*s*) is the z-score for sequence *s* and compound *drug*, *δ*_*drug*_(*s*) is the DES for sequence *s* and compound *drug*, *μ*_*drug*_ is the mean DES value calculated with the Naïve_PRRT_ or Naïve_IN_ datasets, and *σ*_*drug*_ is the corresponding standard deviation.

#### 4.8.2. Estimation of cutoffs of drug-exposure scores

Two sets of cutoffs were determined for each final DES model. The following goals are addressed by each set of cutoffs: (1) prediction of drug exposure and (2) prediction of drug resistance. Each set of cutoffs includes a lower and an upper cutoff for the corresponding DES models. The description of the methods used for determination of these cutoffs follows.

#### 4.8.3. Cutoffs maximizing the performance of the prediction of drug exposure (DEMax cutoffs)

ExposurePheno_*drug*_ cross-validation sets were interpreted with the corresponding final DES models that were trained on them (this is also called calculation of reinsertion predictions). For each cross-validation set, an upper and a lower cutoff were estimated such that the AUC of the drug-exposure prediction is maximized. We call these cutoffs the DEMax cutoffs, and they allow for the discretization of a DES for a drug into the categories *unexposed* (U), *possible exposure* (PE) and *probably exposed* (E). A detailed description of the procedure with which DEMax cutoffs were determined follows.

Function (1) was defined for discretization of a value *δ*_*s*_ ∈ ℝ associated to a sequence s by using the lower and upper cutoffs *c*_*L*_, *c*_*U*_ *∈ ℝ*.

$$\text{discretize}\left(c_{\text{L}},\;c_{\text{U}},\delta_{s}\right)=\{\begin{array}{ll} & 1,\;\text{if}\;\delta_{s}<c_{\text{L}}\\ 2, & \text{if }c_{\text{L}}\leq \;\;\delta_{s}\;\leq c_{\text{U}}\\ & 3,\;\text{if}\;c_{\text{U}}<\;\;\delta_{s} \end{array}$$ (2)

Let *Δ*_*drug*_ ∈ ℝ^*n*^ be a vector of DES predicting the drug exposure of each of *n* sequences *s* to *drug*, and let *E*_*drug*_ ∈ {0,1}^n^ be the corresponding vector of class labels, indicating whether each sequence *s* was exposed to the drug or not. Application of cutoffs *c*_*L*_, *c*_*U*_ and Function (2) to a vector of DES *Δ*_*drug*_ results in the discrete DES vector discretize(*c*_*L*_, *c*_*U*_, *Δ*_*drug*_). For each bootstrap replicate, an upper and a lower cutoff *c*_*L*_ and *c*_*U*_ were determined as

$$\underset{c_{L},\;c_{U}}{\text{argmax}}\text{ AUC}\left(\text{discretize}\left(c_{\text{L}},\;c_{\text{U}},\;\Delta_{drug}\right),\;E_{drug}\right)\;,$$ (3)

where AUC(discretize(*c*_*L*_, *c*_*U*_, *Δ*_*drug*_), *E*_*drug*_) is the AUC quantifying the performance of discretize(*c*_*L*_, *c*_*U*_, *Δ*_*drug*_) in predicting exposure to *drug* for each sequence with a DES in *Δ*_*drug*_. AUC maximization was performed via grid search over *c*_*L*_ and *c*_*U*_.

ExposurePheno_*drug*_ cross-validation sets were interpreted with the corresponding final models that were trained on them. Two thousand bootstrap replicates of the DES of each cross-validation set were created. For each bootstrap replicate and the corresponding class labels, an upper and a lower cutoff were determined by AUC maximization Eq (3). The resulting 2,000 upper and 2,000 lower cutoffs for each final drug-exposure model were averaged to yield the final set of cutoffs. We call these cutoffs the DEMax cutoffs. If a DES for a drug is less than both cutoffs for that drug, then we discretize that DES as *unexposed* (U). If a DES is greater or equal than the lower cutoff, but less or equal than the upper cutoff, we discretize that DES as *intermediate exposure* (IE). Finally, if a DES is greater than both cutoffs, then we discretize that DES as *exposed* (E).

#### 4.8.4. Phenotypically-guided cutoffs for prediction of phenotypic in-vitro drug resistance (pheno cutoffs)

A set of clinically relevant cutoffs for PhenoSense GPPs was obtained from the HIVdb website [14] and is composed as follows. 3TC: 3 and 20; ABC: 3 and 6; AZT: 3 and 10; d4T: 1.5 and 2; ddI: 1.5 and 2; TDF 1.5 and 4; all non-nucleoside reverse-transcriptase inhibitors (NNRTIs): 3 and 10; and all INIs 4 and 20. The set of clinically-relevant cutoffs were used for discretizing PhenoSense GPPs in D_Pheno_ into the categories *susceptible* (S), *intermediate* (I) or *resistant* (R), henceforth called the true labels. The genotypes associated with these GPPs were interpreted with the final DES models. For each drug, an upper and a lower DES cutoff yield predicted GPP labels. These cutoffs, which we call pheno cutoffs, are determined such that the sum of the penalties quantifying the differences between the true labels and the predicted labels is minimized. An individual penalty equals one, if the true label was *R* and the predicted label was *S*. If the true label is *I*, and the predicted label *S*, the penalty equals 0.75. All other differences between true and predicted labels were penalized with the value 0.5, while the equality of true and predicted labels was not penalized. Pheno cutoffs allow for discretization of a DES for a drug as *susceptible* (S), *intermediate* (I) or *resistant* (R). Further details on the cutoff-determination procedure, including the rationale for choosing the penalty values follow.

The error matrix *E* ∈ ℝ^3x3^ Eq (4) was defined for penalizing the misclassification of a discretized value *δ*_*s*_ with label *l ∈ {1*,*2*,*3}* and predicted label l^∈{1,2,3}

$$E_{\left(l,\hat{l}\right)}=\left(\begin{array}{ccc} 0 & 0.5 & 0.5\\ 0.5 & 0 & 0.5\\ 1 & 0.75 & 0 \end{array}\right)$$ (4)

The rationale for choosing the values of the error matrix follows. Diagonal entries are zero, as correct classification incurs no penalty. From a clinical perspective, the worst kind of misclassification that can occur is the classification of a resistant viral strain (label 3) as susceptible (label 1), since the prescription of a therapy including a thus misclassified compound could compromise the susceptibility of all compounds in the therapy. Therefore, this kind of misclassification was assigned the maximum penalty, one. Misclassification of a resistant strain as intermediate (label 2) deserves a smaller penalty, as surpassing the lower cutoff indicates a clinically-relevant decrease in susceptibility, albeit implying that some susceptibility is given. Therefore, this kind of misclassification was assigned the penalty 0.75. All other types of misclassifications are considered equally undesirable, but less severe than the first two, and were assigned the penalty 0.5. Clinically-relevant cutoffs were used to discretize PhenoSense GPPs in D_Pheno_ with Function (2), yielding their labels. The genotypes s associated with these GPPs were interpreted with the DES models. For each drug involved in a GPP, 2,000 bootstrap replicates of the PhenoSense GPPs in D_Pheno_ were sampled. In order to assign to each of the three classes the same weight in this procedure, each bootstrap replicate was constructed using an equal number of GPPs with each label. For each drug, this number was equal to the maximum number of GPPs with a certain label. Each bootstrap replicate was used to determine a lower and an upper cutoff ${\hat{c}}_{L}$,${\hat{c}}_{U}$ which minimizes the sum of the penalties $E_{\left(l,\hat{l}\right)}$ for each label *l* = discretize(*c*_L_, *c*_U_, *RF*_*s*_) with corresponding prediction $\hat{l}=\;\text{discretize}\left({\hat{c}}_{L},\;{\hat{c}}_{U},DES_{s}\right)$ for a resistance factor RF and a drug-exposure score DES associated with genotype s. The resulting 2,000 cutoff pairs for each drug and DES model were averaged, yielding the final phenotypically guided cutoffs. If a DES for a drug is less than both cutoffs for that drug, then we discretize that DES as *susceptible* (S). If a DES is greater or equal than the lower cutoff, but less or equal than the upper cutoff, we discretize that DES as *intermediate* (I). Finally, if a DES is greater than both cutoffs, then we discretize that DES as *resistant* (R).

#### 4.8.5. Extraction of input-feature weights from drug-exposure models

For the purpose of displaying the input features (i.e. HIV-1 substitutions, insertions, and deletions) with the largest influence on a DES interpretation, we represented the SVCs that produce DES as linear functions. Let *x*_*i*_ ∈ {0,1}^*p*^, *i* ∈ {1, …, *n*} be the Support Vectors for a given DES model, *α*_*i*_ ∈ ℝ their corresponding Support-Vector coefficients, and *ρ* ∈ ℝ their intercept. The linear-function representation for a DES model is given by

$$f\left(x_{s}\right)=\overset{n}{\underset{i=1}{\sum }}\alpha_{i}x_{i}x_{s}-\rho ,$$ (5)

where *x*_*s*_ ∈ {0,1}^*p*^ is the encoding for input sequence *s*. Given an encoded sequence *x*_*s*_, the linear Function (5) produces the same numerical output as the corresponding DES SVC. The linear function consists of an offset (also called y-axis intercept) and *p* coefficients that correspond to the components of the vectors that encode each sequence. The vector $\sum_{i=1}^{n}\alpha_{i}x_{i}$ contains these coefficients (also called weights). Since the encoding of the sequence *x*_*s*_ is binary, DES calculation can be performed by adding the offset to the coefficients that correspond to the input features that are present in sequence *s*. In our web service, we display for each drug a selection of features of the input sequence. These features have the largest absolute values of the coefficients in the linear-function representation of DES models. Features with positive coefficients increase DES and are displayed in red. Features with negative coefficients decrease DES and are displayed in green.

## Supporting information

S1 File This file contains one supplementary figure as well as six supplementary tables. (DOC)
