# Supplementary material for: Using drug exposure for predicting drug resistance – A data-driven genotypic interpretation tool
Source: PLoS One. 2017 Apr 10;12(4):e0174992. doi: 10.1371/journal.pone.0174992 (PMC5386274; doi:10.1371/journal.pone.0174992)
Supplement: S1 File — (DOC) [file pone.0174992.s001.doc]

# Supplement of Using Drug Exposure for Predicting Drug Resistance – A Data-Driven Genotypic Interpretation Tool

Alejandro Pironti ^1^*, Nico Pfeifer ^1^, Hauke Walter ^2, 3^, Björn-Erik O. Jensen ^4^, Maurizio Zazzi ^5^, Perpétua Gomes ^6, 7^, Rolf Kaiser ^8^, Thomas Lengauer ^1^

1. Department of Computational Biology and Applied Algorithmics, Max-Planck-Institut für Informatik, Saarbrücken, Germany
2. Medizinisches Infektiologiezentrum Berlin, Berlin, Germany
3. Medizinisches Labor Stendal, Stendal, Germany
4. Clinic for Gastroenterology, Hepatology, and Infectiology, University Clinic of Düsseldorf, Düsseldorf, Germany
5. Department of Medical Biotechnology, University of Siena, Siena, Italy
6. Laboratorio de Biologia Molecular, LMCBM, SPC, HEM - Centro Hospitalar de Lisboa Ocidental, Lisbon, Portugal
7.Centro de Investigacao Interdisciplinar Egas Moniz (CiiEM), Instituto Superior de Ciencias da Saude Sul, Caparica, Portugal
8. Institute for Virology, University Clinic of Cologne, Germany.

* Corresponding author

# Supplementary Figures


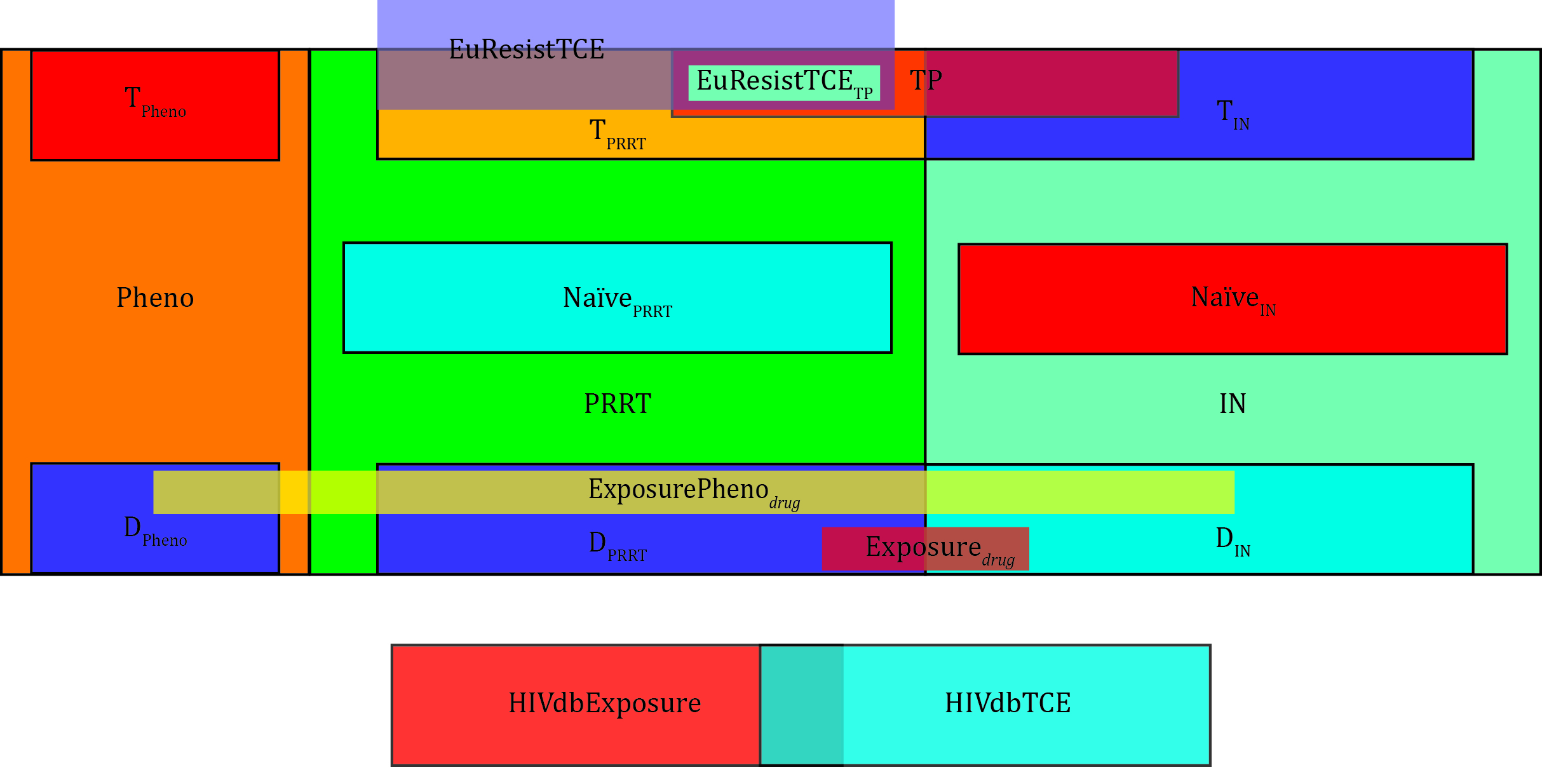


**Fig. A Venn Diagram of Datasets.** The figure above depicts the inclusion relationships between the datasets used in this work, with respect to nucleotide sequences (note that the datasets contain other information in addition to nucleotide sequences). Concise descriptions of the contents of each dataset can be found in Supplementary Table 1. The size of each box in the diagram is not proportional to the size of the datasets.

# Supplementary Tables

**Table A. DEMax Cutoffs.**

|  | **Lower Cutoff (SD)** | **Upper Cutoff (SD)** |
| --- | --- | --- |
| **3TC/FTC** | -0.9 (0) | 0.22 (0.07) |
| **ABC** | -0.8 (0.02) | 0.68 (0.07) |
| **AZT** | -0.9 (0.01) | 0.58 (0.06) |
| **d4T** | -0.77 (0.07) | 0.49 (0.13) |
| **ddI** | -0.83 (0.05) | 0.61 (0.11) |
| **ddC** | -0.69 (0.05) | 0.8 (0.05) |
| **TDF** | -0.75 (0.05) | 0.54 (0.12) |
| **EFV** | -1 (0) | -0.5 (0.07) |
| **ETR** | -0.81 (0.04) | 0.81 (0.15) |
| **DLV** | -0.9 (0.02) | 0.34 (0.24) |
| **NVP** | -0.9 (0) | 0.87 (0.05) |
| **RPV** | -0.49 (0.19) | 0.11 (0.11) |
| **APV/FPV** | -0.9 (0) | 0.79 (0.05) |
| **ATV** | -0.9 (0.01) | 0.46 (0.14) |
| **DRV** | -0.91 (0.03) | 0.34 (0.3) |
| **IDV** | -0.9 (0) | 0.18 (0.12) |
| **LPV** | -0.91 (0.03) | 0.61 (0.33) |
| **NFV** | -1 (0) | -0.08 (0.12) |
| **SQV** | -0.9 (0.02) | 0.55 (0.11) |
| **TPV** | -0.9 (0) | 0.7 (0.08) |
| **RAL** | -0.88 (0.04) | 0.11 (0.13) |
| **EVG** | -0.53 (0.42) | -0.2 (0.06) |
| **Naïve** | −0.51 (0.12) | 0.89 (0.02) |

The final DEMax cutoffs were obtained by averaging the cutoffs obtained with 2000 bootstrap replicates of the cross−validation sets.
SD: Standard Deviation. 3TC: lamivudine, ABC: abacavir, AZT: zidovudine, d4T: stavudine, ddC: zalcitabine, ddI: didanosine, FTC: emtricitabine, TDF: tenofovir, DLV: delavirdine, EFV: efavirenz, ETR: etravirine, NVP: nevirapine, RPV: rilpivirine, APV: amprenavir, ATV: atazanavir, DRV: darunavir, FPV: fosamprenavir, IDV: indinavir, LPV: lopinavir, NFV: nelfinavir, SQV: saquinavir, TPV: tipranavir, EVG: elvitegravir, RAL: raltegravir.

**Table B. pheno Cutoffs.**

|  | **Lower Cutoff (SD)** | **Upper Cutoff (SD)** |
| --- | --- | --- |
| **3TC/FTC** | 0.1 (0.07) | 1.95 (0.11) |
| **ABC** | -0.05 (0.17) | 0.66 (0.27) |
| **AZT** | 0.53 (0.16) | 0.59 (0.16) |
| **d4T** | -0.24 (0.07) | 0.24 (0.1) |
| **ddI** | -0.16 (0.11) | 0.19 (0.29) |
| **TDF** | -0.51 (0.13) | 0.11 (0.46) |
| **EFV** | -0.65 (0.02) | 0.87 (0.08) |
| **ETR** | -0.55 (0.41) | 0.9 (0.02) |
| **DLV** | -0.74 (0.11) | 0.79 (0.14) |
| **NVP** | -0.79 (0.04) | 0.07 (0.25) |
| **RPV** | -0.43 (0.09) | 0.69 (0.22) |
| **APV/FPV** | -0.07 (0.07) | 1.4 (0.17) |
| **ATV** | -0.42 (0.15) | 0.99 (0.05) |
| **DRV** | 0.55 (0.16) | 1.9 (0.02) |
| **IDV** | -0.36 (0.16) | 1.36 (0.08) |
| **LPV** | 0.32 (0.06) | 1.18 (0.08) |
| **NFV** | -0.81 (0.03) | 0.84 (0.04) |
| **SQV** | -0.1 (0.04) | 1.44 (0.08) |
| **TPV** | -0.67 (0.11) | 0.97 (0.02) |
| **RAL** | 0.04 (0.29) | 1.04 (0.06) |
| **EVG** | -0.4 (0.02) | 0.89 (0.01) |

The final pheno cutoffs were obtained by averaging the cutoffs obtained with 2000 bootstrap replicates of the cross−validation sets. SD: Standard Deviation.
3TC: lamivudine, ABC: abacavir, AZT: zidovudine, d4T: stavudine, ddC: zalcitabine, ddI: didanosine, FTC: emtricitabine, TDF: tenofovir, DLV: delavirdine, EFV: efavirenz, ETR: etravirine, NVP: nevirapine, RPV: rilpivirine, APV: amprenavir, ATV: atazanavir, DRV: darunavir, FPV: fosamprenavir, IDV: indinavir, LPV: lopinavir, NFV: nelfinavir, SQV: saquinavir, TPV: tipranavir, EVG: elvitegravir, RAL: raltegravir.

**Table C. Number of Phenotypes by Drug in the Pheno datasets, Clinical Cutoffs.**

|  | **Susceptible** | **Intermediate** | **Resistant** | **Total** | **Susceptible** | **Intermediate** | **Resistant** | **Total** |
| --- | --- | --- | --- | --- | --- | --- | --- | --- |
|  | **D_Pheno_** | | | | **T_Pheno_** | | | |
| **3TC** | 531 | 255 | 760 | 1546 | 57 | 30 | 79 | 166 |
| **ABC** | 656 | 430 | 387 | 1473 | 74 | 49 | 43 | 166 |
| **AZT** | 820 | 207 | 540 | 1567 | 89 | 25 | 51 | 165 |
| **d4T** | 827 | 264 | 482 | 1573 | 92 | 31 | 45 | 168 |
| **ddI** | 735 | 464 | 376 | 1575 | 77 | 48 | 43 | 168 |
| **TDF** | 834 | 300 | 100 | 1234 | 82 | 40 | 10 | 132 |
| **DLV** | 883 | 186 | 569 | 1638 | 89 | 26 | 54 | 169 |
| **EFV** | 918 | 178 | 556 | 1652 | 101 | 12 | 58 | 171 |
| **ETR** | 327 | 88 | 61 | 476 | 37 | 9 | 6 | 52 |
| **NVP** | 794 | 86 | 773 | 1653 | 88 | 8 | 79 | 175 |
| **RPV** | 107 | 36 | 33 | 176 | 14 | 2 | 5 | 21 |
| **ATV** | 541 | 202 | 391 | 1134 | 62 | 26 | 43 | 131 |
| **DRV** | 527 | 71 | 31 | 629 | 65 | 11 | 3 | 79 |
| **FPV** | 1041 | 248 | 406 | 1695 | 117 | 27 | 49 | 193 |
| **IDV** | 859 | 361 | 514 | 1734 | 91 | 45 | 58 | 194 |
| **LPV** | 832 | 296 | 340 | 1468 | 94 | 38 | 40 | 172 |
| **NFV** | 738 | 113 | 929 | 1780 | 75 | 13 | 111 | 199 |
| **SQV** | 1006 | 251 | 484 | 1741 | 108 | 30 | 57 | 195 |
| **TPV** | 575 | 183 | 96 | 854 | 70 | 25 | 11 | 106 |
| **EVG** | 447 | 57 | 94 | 598 | 46 | 9 | 6 | 61 |
| **RAL** | 478 | 66 | 86 | 630 | 48 | 3 | 14 | 65 |

The numbers of phenotypes measured with the PhenoSense assay in the Pheno_D_ and Pheno_T_ datasets are tabulated above by drug. Clinical resistance-factor cutoffs were used for dichotomizing phenotypes into *susceptible*, *intermediate*, and *resistant*.
3TC: lamivudine, ABC: abacavir, AZT: zidovudine, d4T: stavudine, ddC: zalcitabine, ddI: didanosine, FTC: emtricitabine, TDF: tenofovir, DLV: delavirdine, EFV: efavirenz, ETR: etravirine, NVP: nevirapine, RPV: rilpivirine, APV: amprenavir, ATV: atazanavir, DRV: darunavir, FPV: fosamprenavir, IDV: indinavir, LPV: lopinavir, NFV: nelfinavir, SQV: saquinavir, TPV: tipranavir, EVG: elvitegravir, RAL: raltegravir.

**Table D. Performance of Prediction of Drug-Exposure (AUC) on T_PRRT_ , T_IN_, TP and HIVdbExposure with Discrete Output.**

|  | **T_PRRT_, T_IN_** | **TP** | **HIVdbExposure** |
| --- | --- | --- | --- |
| **3TC/FTC** | 0.83 | 0.41 | 0.59 |
| **ABC** | 0.7 | 0.56 | 0.66 |
| **AZT** | 0.81 | 0.61 | 0.56 |
| **d4T** | 0.82 | 0.6 | 0.6 |
| **ddC** | 0.8 | 0.57 | NA |
| **ddI** | 0.82 | 0.64 | 0.68 |
| **TDF** | 0.68 | 0.5 | 0.62 |
| **DLV** | 0.79 | 0.57 | NA |
| **EFV** | 0.73 | 0.53 | 0.8 |
| **ETR** | 0.73 | 0.77 | 0.94 |
| **NVP** | 0.74 | 0.59 | 0.77 |
| **APV/FPV** | 0.77 | 0.55 | 0.78 |
| **ATV** | 0.63 | 0.5 | 0.6 |
| **DRV** | 0.67 | 0.58 | 0.89 |
| **IDV** | 0.76 | 0.6 | 0.74 |
| **LPV** | 0.71 | 0.56 | 0.64 |
| **NFV** | 0.73 | 0.6 | 0.71 |
| **SQV** | 0.79 | 0.59 | 0.74 |
| **TPV** | 0.82 | 0.74 | 0.93 |
| **RAL** | 0.7 | 0.30 | NA |
| **Naïve** | 0.87 | NA | NA |
| **Mean (SD)** | 0.76 (0.06) | 0.57 (0.1) | 0.72 (0.12) |

Drug-exposure prediction performances on sequences in the T_PRRT_, T_IN_, TP and HIVdbExposure datasets were calculated after DES discretization with DEMax cutoffs. Only ExpsourePheno models were used.
NA: Not available; SD: Standard deviation. 3TC: lamivudine, ABC: abacavir, AZT: zidovudine, d4T: stavudine, ddC: zalcitabine, ddI: didanosine, FTC: emtricitabine, TDF: tenofovir, DLV: delavirdine, EFV: efavirenz, ETR: etravirine, NVP: nevirapine, RPV: rilpivirine, APV: amprenavir, ATV: atazanavir, DRV: darunavir, FPV: fosamprenavir, IDV: indinavir, LPV: lopinavir, NFV: nelfinavir, SQV: saquinavir, TPV: tipranavir, EVG: elvitegravir, RAL: raltegravir.

**Table E. Misclassification Rates of SIR-Discretized PhenoSense Genotype-Phenotype Pairs when Predicted with Drug-Exposure Scores with pheno Discretization.**

| **ExposurePheno** | | | | | | | | | | |
| --- | --- | --- | --- | --- | --- | --- | --- | --- | --- | --- |
|  | **M** | **M_S_** | **M_I_** | **M_R_** | **S→I** | **S→R** | **I→S** | **I→R** | **R→S** | **R→I** |
| **3TC/ FTC** | 0.31 | 0.09 | 0.1 | 0.13 | 0.22 | 0.04 | 0.23 | 0.3 | 0 | 0.27 |
| **ABC** | 0.35 | 0.1 | 0.19 | 0.06 | 0.11 | 0.13 | 0.24 | 0.39 | 0.02 | 0.19 |
| **AZT** | 0.35 | 0.19 | 0.15 | 0.01 | 0.02 | 0.33 | 0.16 | 0.84 | 0.04 | 0 |
| **d4T** | 0.39 | 0.2 | 0.16 | 0.03 | 0.19 | 0.18 | 0.13 | 0.71 | 0.09 | 0.02 |
| **ddI** | 0.41 | 0.13 | 0.25 | 0.04 | 0.03 | 0.25 | 0.17 | 0.69 | 0.1 | 0.05 |
| **TDF** | 0.52 | 0.27 | 0.24 | 0.02 | 0.18 | 0.26 | 0.18 | 0.6 | 0 | 0.2 |
| **DLV** | 0.2 | 0.08 | 0.08 | 0.04 | 0.15 | 0.01 | 0.08 | 0.48 | 0.04 | 0.07 |
| **EFV** | 0.14 | 0.06 | 0.04 | 0.04 | 0.07 | 0.03 | 0.25 | 0.25 | 0.02 | 0.1 |
| **ETR** | 0.28 | 0.18 | 0.08 | 0.02 | 0.23 | 0.03 | 0 | 0.44 | 0 | 0.17 |
| **RPV** | 0.32 | 0.16 | 0.05 | 0.11 | 0.25 | 0 | 0 | 0.5 | 0.2 | 0.2 |
| **NVP** | 0.19 | 0.13 | 0.04 | 0.03 | 0.2 | 0.06 | 0.25 | 0.5 | 0.04 | 0.03 |
| **APV/ FPV** | 0.25 | 0.14 | 0.06 | 0.05 | 0.21 | 0.03 | 0.07 | 0.37 | 0 | 0.18 |
| **ATV** | 0.21 | 0.06 | 0.08 | 0.06 | 0.11 | 0.02 | 0.08 | 0.35 | 0 | 0.19 |
| **DRV** | 0.14 | 0.05 | 0.08 | 0.01 | 0.06 | 0 | 0.45 | 0.09 | 0 | 0.33 |
| **IDV** | 0.27 | 0.1 | 0.1 | 0.06 | 0.21 | 0.01 | 0.04 | 0.4 | 0 | 0.21 |
| **LPV** | 0.22 | 0.07 | 0.13 | 0.02 | 0.11 | 0.02 | 0.05 | 0.53 | 0 | 0.08 |
| **NFV** | 0.16 | 0.04 | 0.03 | 0.1 | 0.11 | 0 | 0.08 | 0.31 | 0 | 0.17 |
| **SQV** | 0.28 | 0.12 | 0.06 | 0.1 | 0.22 | 0 | 0.13 | 0.27 | 0 | 0.33 |
| **TPV** | 0.23 | 0.11 | 0.08 | 0.03 | 0.17 | 0 | 0.16 | 0.2 | 0 | 0.27 |
| **RAL** | 0.15 | 0.05 | 0.06 | 0.05 | 0.04 | 0.02 | 0.25 | 0.75 | 0 | 0.23 |
| **EVG** | 0.2 | 0.03 | 0.12 | 0.05 | 0.04 | 0 | 0.22 | 0.56 | 0 | 0.6 |
| **Mean (SD)** | 0.27 (0.1) | 0.11 (0.06) | 0.1 (0.06) | 0.05 (0.03) | 0.14 (0.08) | 0.07 (0.1) | 0.15 (0.11) | 0.45 (0.19) | 0.03 (0.05) | 0.19 (0.14) |

The misclassification rate (M) was calculated as the fraction of discordant SIR label pairs obtained with PhenoSense genotype-phenotype pairs (GPPs) and drug-exposure scores (DES). The misclassification rates M_S_, M_I_, and M_R_ denote the fraction of misclassified *susceptible*-, *intermediate*-, and *resistant*-labeled GPPs, respectively. The R to S (R**→**S) misclassification rate states the fraction of *resistant*-labeled GPPs which were predicted *susceptible*. Conversely, the S to R (S**→**R) misclassification rate states the fraction of *susceptible*-labeled GPPs which were predicted *resistant.*
SD: Standard deviation. 3TC: lamivudine, ABC: abacavir, AZT: zidovudine, d4T: stavudine, ddC: zalcitabine, ddI: didanosine, FTC: emtricitabine, TDF: tenofovir, DLV: delavirdine, EFV: efavirenz, ETR: etravirine, NVP: nevirapine, RPV: rilpivirine, APV: amprenavir, ATV: atazanavir, DRV: darunavir, FPV: fosamprenavir, IDV: indinavir, LPV: lopinavir, NFV: nelfinavir, SQV: saquinavir, TPV: tipranavir, EVG: elvitegravir, RAL: raltegravir.
